# S100B and APP Promote a Gliocentric Shift and Impaired Neurogenesis in Down Syndrome Neural Progenitors

**DOI:** 10.1371/journal.pone.0022126

**Published:** 2011-07-11

**Authors:** Jie Lu, Giuseppe Esposito, Caterina Scuderi, Luca Steardo, Laurent C. Delli-Bovi, Jonathan L. Hecht, Bryan C. Dickinson, Christopher J. Chang, Takashi Mori, Volney Sheen

**Affiliations:** 1 Department of Neurology, Beth Israel Deaconess Medical Center, Harvard Medical School, Boston, Massachusetts, United States of America; 2 Department of Human Physiology and Pharmacology “V. Erspamer”, Faculty of Pharmacy, University of Rome “La Sapienza”, Piazzale Aldo Moro, Rome, Italy; 3 Department of Obstetrics and Gynecology, Brigham and Women's Hospital, Harvard Medical School, Boston, Massachusetts, United States of America; 4 Department of Pathology, Beth Israel Deaconess Medical Center, Harvard Medical School, Boston, Massachusetts, United States of America; 5 Howard Hughes Medical Institute, Department of Chemistry, University of California, Berkeley, California, United States of America; 6 Howard Hughes Medical Institute, University of California, Berkeley, California, United States of America; 7 Departments of Biomedical Sciences and Pathology, Saitama Medical Center and Saitama Medical University, Kawagoe, Saitama, Japan; Hôpital Robert Debré, France

## Abstract

Down syndrome (DS) is a developmental disorder associated with mental retardation (MR) and early onset Alzheimer's disease (AD). These CNS phenotypes are attributed to ongoing neuronal degeneration due to constitutive overexpression of chromosome 21 (HSA21) genes. We have previously shown that HSA21 associated S100B contributes to oxidative stress and apoptosis in DS human neural progenitors (HNPs). Here we show that DS HNPs isolated from fetal frontal cortex demonstrate not only disturbances in redox states within the mitochondria and increased levels of progenitor cell death but also transition to more gliocentric progenitor phenotypes with a consequent reduction in neuronogenesis. HSA21 associated S100B and amyloid precursor protein (APP) levels are simultaneously increased within DS HNPs, their secretions are synergistically enhanced in a paracrine fashion, and overexpressions of these proteins disrupt mitochondrial membrane potentials and redox states. HNPs show greater susceptibility to these proteins as compared to neurons, leading to cell death. Ongoing inflammation through APP and S100B overexpression further promotes a gliocentric HNPs phenotype. Thus, the loss in neuronal numbers seen in DS is not merely due to increased HNPs cell death and neurodegeneration, but also a fundamental gliocentric shift in the progenitor pool that impairs neuronal production.

## Introduction

Down syndrome (DS) arises from a triplication of genes on chromosome 21 (HSA21) and is characterized by neurological complications including mental retardation and early onset Alzheimer's disease (AD) [1]. The reduced brain size and simplified gyral patterning are thought to be major determinants of the cognitive impairment in DS individuals. At the cellular level, DS brains show prolongation in the cell cycle length of neural progenitors [2], [3], as well as increased oxidative stress and mitochondrial dysfunction within neurons [3], [4], [5]. These findings would suggest that both abnormalities in proliferation and progressive neuronal loss through apoptosis contribute to the developmental neuropathology in DS.

Studies using DS human neural progenitors (HNPs) have proposed several mechanisms underlying the loss in neuronal numbers in DS brain. Expression profiling of 18 week gestational age (W GA) DS HNPs followed by in vitro studies are able to demonstrate constitutive overexpression of HSA21 associated S100B, leading to increased reactive oxygen species (ROS) formation, activation of stress response kinases, and cell death [6]. Comparison of the differentially expressed genes in DS versus wild type (WT) HNPs at 13W GA demonstrates that progenitors exhibited impairments in interneuron neurogenesis, related to increased expression of the transcription factor COUP-TF1/NR2F1 and downregulation of the interneuron related genes DLX1, DLX2 and DLX5 [7]. Finally, other studies have reported a decrease in neuroectodermal genes such as Nestin and Tubb3 in DS HNPs with a corresponding increase in mesodermal genes such as Snail1 and Pitx2, indicating that HSA21 genes such as DYRK1A could regulate various embryonic lineages [8].

Interactions between HSA21-associated S100B and amyloid precursor protein (APP) might effect neural progenitor development and contribute to the cognitive impairment in DS. Recent studies have shown deleterious effects from the constitutive overexpression of the HSA21-associated S100B in DS HNPs. Soluble S100B activates the receptor for advanced glycation endproducts (RAGE), leading to generation of reactive oxygen species (ROS), and induction of MAP kinases, including JNK. JNK activation induces Dickopff-1 expression that in turn promotes GSK3ß phosphorylation and tau hyperphosphorylation [6], [9]. The HSA21 associated gene APP contributes to the pathological deposition of beta amyloid (Aβ) in the brain [10]. Amyloid-forming proteins such as Aβ both accelerate tau hyperphosphorylation and represent a second group of RAGE ligand that could further enhance S100B-mediated cell injury [11]. These observations raise the possibility that these two contiguous genes on HSA21 might influence DS progenitor survival and proliferation through a common shared pathway.

Here we show that constitutive overexpression of HSA21 associated S100B and APP promotes a deleterious, cyclical pathway involving synergistic overproduction and hypersecretion of both proteins, altered mitochondrial redox states, cell injury and neuronal death. The ongoing neural injury and inflammation further promote a gliocentric progenitor phenotype and indicate that DS HNPs inherently differ from their normal age-matched counterparts. The gliocentric shift coincides with a decline in neurogenesis. This study describes a potential paradigm whereby early changes in progenitor survival and phenotype could contribute to and explain some of the underlying mechanisms giving rise to the proliferative changes and impaired neuronal production seen in the DS brain.

## Results

### Increased mitochondrial dysfunction, apoptosis, and gliocentric progenitor pool shift in DS fetal brain and HNPs

**Figure 1:**
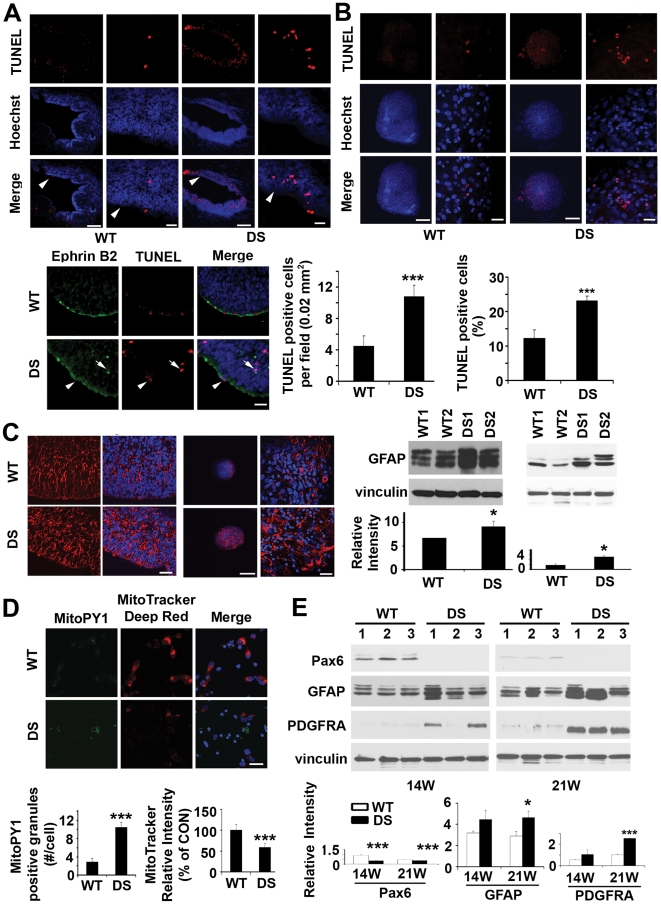
Increased apoptosis, gliosis, mitochondria dysfunction and gliocentric cell fate shift in DS HNPs. (A) Confocal fluorescence photomicrographs demonstrate increased numbers of cells undergoing apoptosis as observed by TUNEL stain (rhodamine, counterstained with Hoechst 33342) along the VZ for DS fetal cortex (18W GA, noted by white arrowheads). Higher magnification images are to the right. The relative number of TUNEL labeled cells is quantified for both WT and DS VZ. (n = 4 for each). Many of the TUNEL positive cells (rhodamine) express the ephrinb2 neural progenitor marker (fluorescein) along the VZ of 18W GA fetal cortex (see white arrowheads in lower panel). (B) The increase in programmed progenitor cell death seen *in vivo* is also appreciated *in vitro* in human DS neurospheres after one week of culture. Quantification of TUNEL-positive cells is below (n>5 neurospheres in each experimental sample). (C) Fluorescent photomicrographs demonstrate increased intensity of immunostaining (rhodamine, counterstained with Hoechst 33342) for GFAP along the VZ of fetal 18W GA DS brain compared to normal age-matched controls. Western blot confirms the increase. The upregulation of GFAP (rhodamine) is also found in the DS neurospheres derived from the VZ of 18W GA fetuses after one week of culture, as shown by immunostaining and western blot. (D) Fluorescence photomicrographs by confocal microscopy demonstrate increased intracellular mitochondrial H_2_O_2_ production showed by MitoPY1 (fluorescein) staining and disrupted mitochondrial membrane potential showed by decreased MitoTracker deep red staining (rhodamine, counterstained with Hoechst 33342) within DS HNPs compared to WT controls (18W GA) after 24 hours of culture. MitoPY1 localizes to the mitochondria and directly assays H_2_O_2_ levels in the organelle. The quantification graphs are showed below. (E) Western blots demonstrate decreased neuroprogenitors shown by pax6 and increased glioprogenitors showed by GFAP and PDGFRA staining in human DS frontal cortex (n = 3 age-matched control and DS fetal tissues, 14W and 21W GA). Quantification is showed below. Scale bars are 200 µm for low magnification and 25 µm for high magnification in A, B and C, 25 µm for D. Data are represented as mean +/− STDEV, * p-value<0.05, ** p-value<0.01, *** p-value<0.001 by two tailed t-test.

While increased ROS, apoptosis and gliosis have been implicated in postnatal DS neurons [3], [12], [13], [14], few studies have addressed whether the same endophenotypes are apparent during cortical development. Our prior expression profiling studies and network analyses suggest that dysregulated genes in DS HNPs form functional clusters involved in redox states, cell death, and glial characteristics [6]. To test these initial observations formally, we asked whether these endophenotypes could be identified in both tissue and HNPs from 14–21W GA DS frontal cortex. There was a two-fold increase of apoptosis by TUNEL labeling along the ventricular and subventricular zones (VZ/SVZ) in multiple 18W GA DS frontal cortex. (**Figure 1A**). Several apoptotic cells expressed ephrinB2 (**Figure 1A** **, see lower panel, white arrows**), a marker for a subset of neural progenitors. Consistent with the observation of increased vulnerability in DS HNPs in vivo, this same accelerated rate of cell death could be appreciated *in vitro* with neurospheres cultured from multiple DS HNP lines, generated from 18W GA DS frontal cortex (**Figure 1B**). We also observed an increase of GFAP expression in both DS fetal VZ/SVZ tissue and HNPs of the same aged frontal cortices (**Figure 1C**). To address the possible mitochondrial involvement leading to the increased cell death and presumed glial progenitor inflammatory response, we used a dual *in situ* labeling technique that incorporated the MitoTracker Deep Red dye (a marker of mitochondrial membrane potential) and MitoPY1 fluorescein dye (a marker of mitochondrial H_2_O_2_ levels) [15] to simultaneously track mitochondrial function and oxidative stress. Compared to WT controls, the DS HNPs showed an increase of H_2_O_2_ production at the mitochondria and simultaneous decrease of mitochondrial membrane potential (**Figure 1D**). The mitochondrial dysfunction, cell death, and adoption of more gliocentric phenotypes in DS HNPs raised the possibility of impaired neuronogenesis over time. We therefore examined the protein expression for several neuronal and glial markers in 14W and 21W GA fetal frontal cortex from both DS and WT controls. The neuronal-restricted progenitor marker Pax6 [16] was strongly expressed in 14W WT HNPs but absent in the 14W DS HNPs. Appropriately at 21W GA (the end of corticogenesis), this marker was still detectable in WT HNPs although at lower levels, but still absent in the DS samples. We also found increased expression for the glial progenitor markers GFAP and PDGFRA [17] in the DS HNPs at both ages, relative to the WT HNPs (**Figure 1E**). The cell fate change in DS was further confirmed by withdrawing growth factor support for the HNPs and inducing differentiation of these progenitors. Fluorescent immunostaining for different cell fate markers and quantification of positively stained cells indicated that DS HNPs underwent increased astrocytic and oligodendrocytic differentiation (with a corresponding reduction in neurogenesis), as compared with control HNPs (**Figure S1**). In summary, these findings suggest that DS HNPs exhibit increased oxidative stress and loss in membrane potential at the mitochondria, undergo an increased rate of apoptosis, and show increased gliocentric characteristics at the expense of neuronal phenotypes.

### Elevated APP and S100B expression, reciprocal regulation, and secretion in DS HNPs

**Figure 2:**
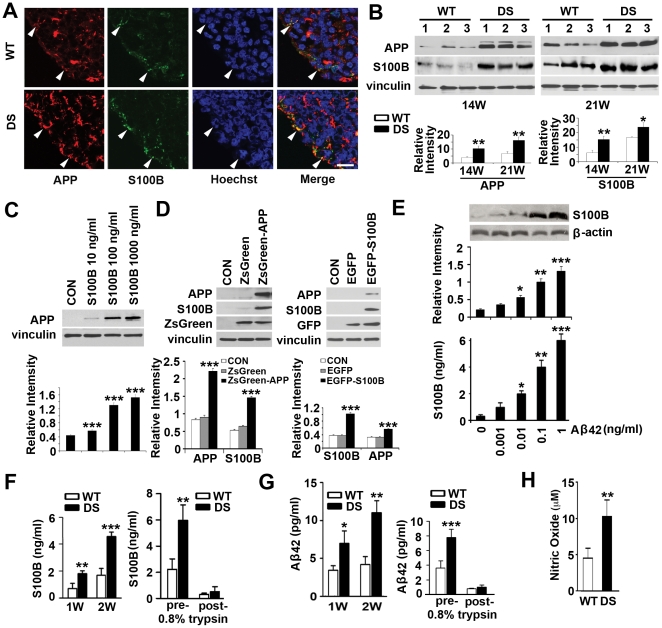
Reciprocal up-regulation of secreted S100B, APP and oxidative stress in DS HNPs. (A) Fluorescent photomicrographs demonstrate increased intensity of staining for both APP (rhodamine) and S100B (fluorescein, counterstained with Hoechst 33342) within DS neural progenitors along the VZ of the 18W GA fetal cortex compared to normal age-matched controls. White arrowheads show the colocalizations of APP and S100B along the VZ. (B) Western blot analyses confirm upregulation of both these proteins within the cortex of the DS brains of multiple independent samples at an age dependent manner (n = 3 age-matched control and DS fetal tissues, 14W and 21W GA). (C) Incubation of S100B (10–1000 ng/ml) for 24 hours in normal cultured HNPs dose-dependently increases APP levels; (D) S100B levels in the cytoplasm are increased with APP overexpression for 4 days, and vice versa, as shown by western blot. (E) Western blot and ELISA assay show a dose-dependent increase in S100B expression and secretion into the culture medium after Aβ42 stimulation for 24 hours. (F) Longer term (1–2 weeks) culturing of DS HNPs results in a progressive increase in the expression of S100B. The increased levels of S100B are largely due to soluble, extracellular S100B as trypsin treatment of the media can degrade the protein. (G) A similar increase in Aβ42 levels is appreciated in DS HNPs cultured over time. Increased levels of Aβ42 are also largely due to soluble, secreted protein that is degraded with trypsin treatment. Prior findings have shown that S100B or amyloid can lead to increased ROS generation. Consistent with these findings, there is an increase of oxidative stress in DS HNPs, as showed by nitric oxide assays (H). Scale bars are 12.5 µm in A; data are represented as mean +/− STDEV, * p-value<0.05, ** p-value<0.01, *** p-value<0.001 by two tailed t-test.

Prior studies have shown that both HSA21-associated APP and S100B levels increase in adult DS brain and that both these proteins might play some role in DS progenitor development [18], [19]. Secretion of either APP or S100B has also been thought to be neuroprotective at low concentrations but neurotoxic at high concentrations [20], [21]. These observations led to the possibility that our novel observation of ongoing progenitor cell death seen early in DS cortical development might be influenced by APP and S100B either through activation of intracellular cell death pathways or secretion of these soluble proteins, causing toxicity to neighboring progenitors. We first examined whether upregulation and colocalization of S100B and APP expression were apparent in the VZ/SVZ regions of human DS fetal cortices. Immunostaining for both S100B and APP showed overlapping, elevated expression of both these proteins within progenitors along the lateral ventricles of 18W GA forebrain (**Figure 2A**). The upregulations of these two proteins in human DS cortex were also appreciated by western blot analyses with a progressive increase seen in 14W and 21W GA DS frontal cortex (**Figure 2B**). Second, normal HNPs treated with soluble S100B protein showed increased APP levels (**Figure 2C**) at concentrations (10ng/ml) comparable to the levels secreted by DS HNPs (**Figure 2F**). Similarly, overexpression of APP within HNPs through lentiviral infection showed a consequent increase of cytoplasmic S100B by western blot, and conversely transient overexpression of S100B within HNPs demonstrated enhanced expression in APP (**Figure 2D**). Furthermore, stimulation of HNPs with Aβ42 caused a consequent dose-dependent increase of S100B expression showed by western blot or soluble S100B secretion into the culture media showed by ELISA (**Figure 2E**), suggestive of a reciprocal synergistic effect. In these studies, we used the ZsGreen-APP lentivirus (10 µl) and EGFP-S100B (5 µg) at doses (**Figure S2A, B**) comparable to the levels of S100B and Aβ42 secreted by DS HNPs (**Figure 2F, G**). Third, Aβ42 and Aβ40 are the two most common products of APP processing, but Aβ42 is the more fibrillogenic and thus associated with disease states [22]. We found that S100B and Aβ42 levels progressively increased within the media of cultured DS progenitors by ELISA analyses and addition of trypsin to the media led to degradation of these proteins, indicating that secretion of the proteins likely contribute to effects on cell viability [23] (**Figure 2F, G**). Fourth, S100B has previously been shown to induce neuronal death through nitric oxide [24] and we found that DS progenitors exhibited higher levels of nitric oxide (**Figure 2H**). Collectively, these series of experiments indicate that constitutive overexpression of HSA21-associated S100B and APP/Aβ42 could promote the pathological expression of the other protein. Secretion of these proteins into the local extracellular milieu even at fairly low levels (as compared to levels required to induce neurotoxicity) in DS HNPs would provide a basis for the observed increase in mitochondrial ROS generation, apoptosis and gliocentric shift.

### Elevated mitochondrial hydrogen peroxide, decreased mitochondrial membrane potential and increased apoptosis in HNPs due to S100B and APP

**Figure 3:**
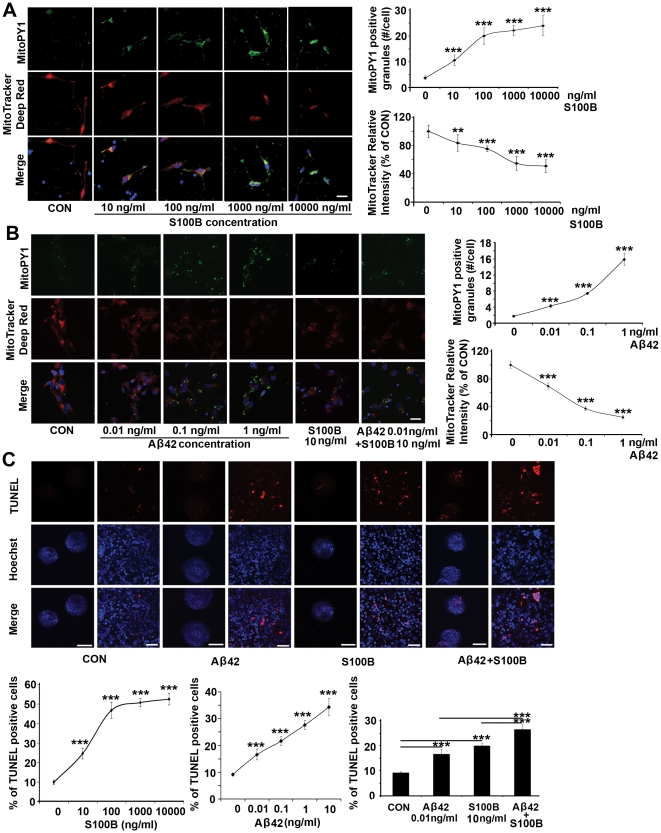
Soluble S100B or Aβ42 treatment promotes mitochondrial H_2_O_2_ production, loss in mitochondrial membrane potential and apoptosis in normal cultured HNPs. (A) S100B exposure for 24 hours dose-dependently increases intracellular mitochondrial H_2_O_2_ production within HNPs, as shown by MitoPY1 (fluorescein, counterstained with Hoechst 33342) staining. MitoPY1 localizes to the mitochondria and directly assays H_2_O_2_ levels in the organelle. S100B treatment also leads to mitochondrial dysfunction in a dose-dependent fashion, as showed by decreased MitoTracker deep red staining (rhodamine). Results are quantified graphically to the right. (B) A similar trend of increased intracellular mitochondrial H_2_O_2_ and decreased mitochondrial membrane function appears after exposure to soluble Aβ42 for 24 hours. The quantification graphs for additive effects of S100B and Aβ42 are showed in **Figure S3E**. (C) Exposure to S100B or Aβ42 at concentrations comparable to that seen in DS HNPs for 24 hours causes apoptosis (showed by TUNEL staining) in WT cultured HNPs. The graphs below show a dose-dependent increase in apoptosis after S100B, APP or both S100B and APP stimulation (n>5 neurospheres in each experimental trial with at least 3 replicates). Scale bars are 25 µm for A and B; 200 µm for low magnification and 25 µm for high magnification in C; data are represented as mean +/− STDEV, *** p-value<0.001 by two tailed t-test and one-way ANOVA.

Elevated levels of secreted S100B or Aβ42 would allow for the proposed paracrine effects in neural progenitors- namely S100B oversecretion leading to RAGE activation and mitochondrial dysfunction with resulting ROS generation, and GSK3ß and tau hyperphosphorylation [6]. APP could additively enhance this pathological pathway by promoting S100B secretion and impairing mitochondrial function. To address these possibilities, we first transfected or infected normal HNPs with either a S100B construct or an APP lentivirus in a dose dependent fashion and observed a corresponding increase in S100B and Aβ42 levels within the cell medium. Furthermore, increases in S100B/Aβ42 in turn caused a decrease in mitochondrial function (**Figure S2A, B**). Concurrent overexpression of both S100B and APP led to a larger decline in mitochondrial function, suggestive of an additive effect (**Figure S2C**). Given that S100B and Aβ42 were secreted, we examined whether exposure of HNPs to varying concentrations of soluble S100B or Aβ42 led to a dose dependent decline in mitochondrial function. We used a dual *in situ* labeling of MitoTracker Deep Red and MitoPY1 fluorescein dyes to track simultaneously mitochondrial function and oxidative stress after S100B/Aβ42 treatment. Increasing concentrations of soluble S100B or Aβ42 resulted in dose dependent declines in mitochondrial respiration, as shown by the loss in rhodamine intensity, and enhanced mitochondrial oxidative stress, as shown by the gain in fluorescein intensity (**Figure 3A, B**). Concurrent S100B and Aβ42 treatment also led to an additive decline in mitochondrial membrane potential and increase in ROS (**Figure S3E**). Because S100B and APP dependent loss of mitochondrial activity does not necessary result in cell death, we wanted to determine the functional outcome of the mitochondrial impairment due to increased levels of these proteins. Thus, we examined the consequent effects of soluble S100B or Aβ42 on HNP viability. We found a dose-dependent increase in apoptosis with overexpression of intracellular APP or S100B through lentiviral infection or transient transfection into control HNPs (**Figure S3A, B**). Again, co-overexpression of these proteins led to additive effects (cell death) and the increase in cell death was not limited to transfected cells (**Figure S3C**). Given our observations of S100B and Aβ42 secretion into the cell medium and the induction of cell death with non-infected/transfected progenitors, we then asked whether exposure of HNPs to progressively higher concentrations of soluble S100B or Aβ42 caused apoptosis. We observed a dose-dependent increase in TUNEL labeling within HNPs following incubation in the respective proteins (**Figure 3C**). To assess whether the *in vitro* observations were similarly appreciated *in vivo*, we examined the cortex of early postnatal (P0) trisomy 16 (Ts65Dn) mice, which have an extra copy of mouse APP but not S100B. We found increased apoptosis in neural progenitors and neurons within the early postnatal cortex of the Ts65Dn mice, albeit to a lesser degree than in the human fetal cortex (**Figure S3D**). To examine more directly the potential synergistic effects of APP and S100B, we quantified the levels of ongoing cell death in the hippocampus of APP (Tg2576) and APP/S100B (Tg2576-huS100B) transgenic adult mice (19 months), and more specifically the subgranular zone of hippocampus. Postnatally, this area is one of the few regions in the central nervous system where neural progenitors reside and exhibit continued neurogenesis, and archival tissue from these mice would provide some indication of *in vivo* effects due to these two proteins. We again appreciated enhanced cell death with overexpression of both, as opposed to overexpression of the individual proteins (**Figure S3D**). Taken in sum, these observations provide a direct causal link between the constitutive overexpression of HSA21-associated S100B and APP (Aβ42) and observed increase in mitochondrial ROS, decline in membrane potentials and increase in apoptosis during neural progenitor development.

### S100B and APP/Aβ42 promote gliocentric phenotypes in HNPs and transgenic mice

**Figure 4:**
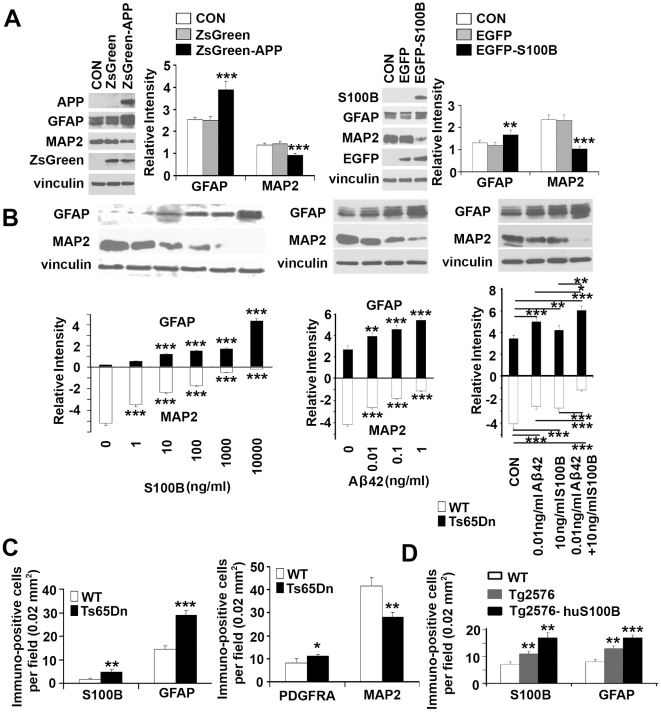
Gliocentric shift due to S100B and APP/Aβ42 in DS HNPs. (A) Lentiviral infections of ZsGreen-APP or transfections of EGFP-S100B constructs into normal HNPs for 4 days promote GFAP and inhibit MAP2 expression, as shown by western blot. (B) Pretreatment of normal, cultured HNPs with increasing concentrations of soluble S100B or Aβ42 for 24 hours shows a dose-dependent increase in GFAP and decrease in MAP2 expression. Co-treatment with S100B and Aβ42 for 24 hours leads to an additive increase in GFAP and decrease in MAP2 expression levels. (C) Quantification graphs from fluorescent photomicrographs (**Figure S4A**) in the cortex of early postnatal (P0) Ts65Dn mice show increased numbers of immunostaining on glial markers such as S100B, GFAP and PDGFRA, and decreased numbers of neuronal staining with MAP2 compared to WT (n = 3 for each group of mice). A similar increase appears in APP (Tg2576) or APP/S100B (Tg2576-huS100B) overexpressing mice compared to age-matched WT control (**Figure S4B** and **S4C**). Data are represented as mean +/− STDEV, * p-value<0.05, ** p-value<0.01, *** p-value<0.001 by two tailed t-test and one-way ANOVA.

Previous reports have implicated potential synergistic effects between various RAGE ligands such as S100B and APP, leading to gliosis [25]. Our prior studies have shown an increase in glial-associated markers S100B and AQP4 within DS HNPs [6], [9]. We have also observed increased glial characteristics within the DS HNP pool, as evidenced by the increased expression of proteins such as GFAP in 18W GA human DS VZ/SVZ and correspondingly, in cultured DS HNPs (**Figure 1B**). The possibility that these altered progenitor phenotypes are due to APP and/or S100B overexpression is supported at several levels. First, HNPs overexpressing APP or S100B exhibited increased GFAP and decreased MAP2 expression (**Figure 4A**). Second, HNPs exposed to S100B or Aβ42 dosed-dependently increased GFAP and decreased MAP2 expression; concurrent exposure of S100B and Aβ42 enhanced GFAP and inhibited MAP2 expression synergistically (**Figure 4B**). Third, the combinatorial effects of APP and S100B in enhancing gliosis, previously appreciated *in vitro*, were partly observed *in vivo*. In the cortical VZ of APP-overexpressing early postnatal (P0) Ts65Dn mice, we observed increased expression of glial markers including S100B and GFAP within the progenitor population along the ventricles of these mice (**Figure 4C** **, S4A**). Increased oligoprogenitor marker PDGFRA and decreased neuronal marker MAP2 expression were also observed in Ts65Dn mice (**Figure S4A**). A similar gliocentric shift was observed in the dentate gyrus of adult (19 months) APP (Tg2576) and APP/S100B (Tg2576-huS100B) transgenic mice with increased expression for GFAP, S100B, CNPase and MBP (**Figure 4D** **, S4B, S4C**). These observations implicate a potential cyclical path of neural progenitor injury whereby overexpression of S100B and APP leads to mitochondrial impairment, cell death and consequent inflammation with a shift toward gliocentric phenotypes. This gliocentric shift would further promote maladaptive responses due to S100B expression and activity, including further accentuation of glial progenitor phenotypes.

### RAGE blockade and APP inhibition in reversal of S100B and APP effects in the DS phenotype

**Figure 5:**
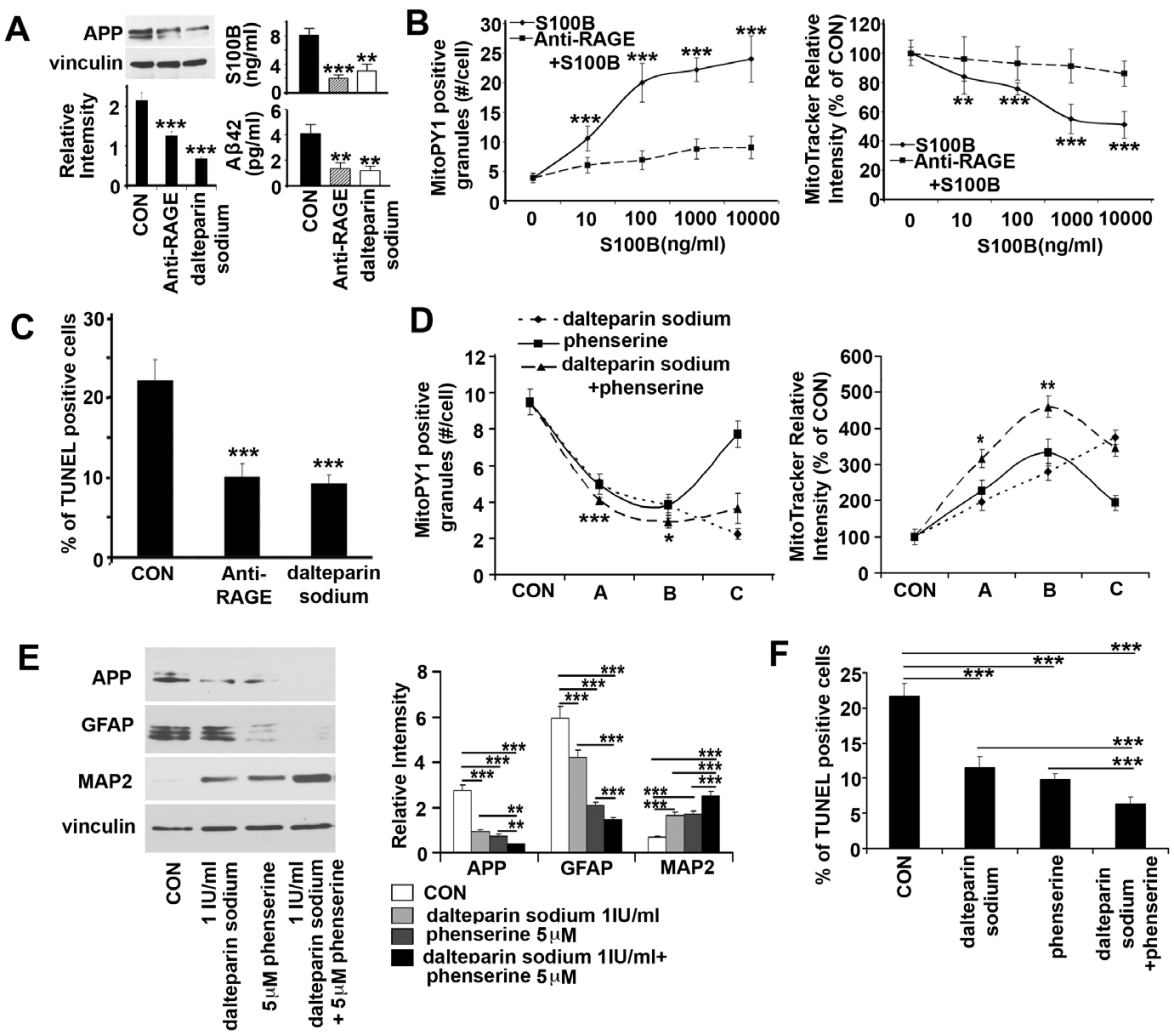
RAGE blocking and APP inhibition synergistically reduce oxidative stress, apoptosis and rescue sequential gliocentric cell fate change in DS HNPs. (A) DS HNPs treated with Anti-RAGE antibody (1 µg/ml) or RAGE antagonist dalteparin sodium (1 IU/ml) for 24 hours show reduced APP expression (left, western blot) and S100B or Aβ42 secretion (right, ELISA). (B) Graphs show the S100B stimulation for 24 hours dose dependently increase H_2_O_2_ production and decrease mitochondrial membrane potential which can be blocked by RAGE antibody (1 µg/ml) or dalteparin sodium (1 IU/ml) (**Figure S5A**). (C) Quantification graph shows the large numbers of TUNEL+ cells in DS HNPs decrease to normal level after Anti-RAGE antibody (1 µg/ml) or dalteparin sodium (1 IU/ml) treatment for 24 hours, and a quantification graph is on the upper left (n>5 neurospheres in each experimental sample) (**Figure S5B**). (D) DS HNPs are treated with dalteparin sodium and phenserine for 24 h, and quantitative analyses of mitochondrial membrane potential and mitochondrial H_2_O_2_ fluorescent intensities are quantified from the photographs (**Figure S5C**) at three separate dosages (A = dalteparin 0.01 IU/ml, phenserine 0.5 µM, or dalteparin 0.01 IU/ml+phenserine 0.5 µM; B = dalteparin 0.1 IU/ml, phenserine 5 µM, or dalteparin 0.1 IU/ml+phenserine 5 µM; C = dalteparin 1 IU/ml, phenserine 50 µM, or dalteparin 1 IU/ml+phenserine 50 µM). The graph shows a synergistic effect of the two drugs with a presumed level of toxicity at the highest concentrations. (E) Pretreatment with phenserine, dalteparin or phenserine+dalteparin for 24 hours reduces GFAP and APP levels but increases MAP2 levels in DS HNPs. Western blot analyses show significant reduction of GFAP and APP and increase of MAP2 expression levels following treatment. Band intensities are graphically quantified below. (F) Quantification graph of TUNEL staining (**Figure S5D**) from DS HNPs treated with dalteparin sodium and phenserine for 24 hours shows decreased TUNEL+ cells compared to controls (n>5 neurospheres in each experimental sample). Data are represented as mean +/− STDEV, * p-value<0.05, ** p-value<0.01, *** p-value<0.001 by one-way ANOVA.

The effects of S100B and APP in DS could be due to disruption of intracellular pathways, secretion and toxicity to neighboring progenitors, or a combination of both mechanisms. We therefore focused on RAGE blockade and APP inhibition to address the contribution of paracrine effects from these proteins. The platelet inhibitor, dalteparin sodium has been shown to have antagonizing effects on RAGE activity [6], [26]. As RAGE is the receptor for S100B and Aβ, we explored the therapeutic efficacy of these agents on several levels. First, treatment of DS HNPs with anti-RAGE antibody or dalteparin sodium resulted in a downregulation of APP expression, as well as S100B and Aβ42 secretion (**Figure 5A**). Second, exposure of control HNPs to various concentrations of soluble S100B (including the pathological dose corresponding to 10 ng/ml secreted by DS HNPs) led to a dose-dependent increase in H_2_O_2_ production as indicated by increasing MitoPY1 fluorescein fluorescence. A similar dose-dependent decrease in mitochondrial membrane potential was seen, corresponding to a decline in MitoTracker deep red rhodamine fluorescence. These pathological changes could be reversed by pretreatment with either RAGE antibody or the RAGE antagonist dalteparin sodium (**Figure 5B** **, S5A**). Third, incubation of DS HNPs with anti-RAGE antibody or dalteparin sodium resulted in an approximate 50% reduction in apoptosis as gauged by TUNEL staining in the neurospheres; the levels were still not back to age-matched WT baseline (**Figure 5C** **, S5B**). Finally, parallel studies were performed to inhibit APP levels using phenserine. Inhibition of the RAGE receptor (dalteparin sodium) and APP (phenserine) provided further HNPs protection, indicating that additional intracellular RAGE-independent mechanisms of cell injury also contribute to the observed phenotypes (**Figure 5D, 5E, 5F** **, S5C, S5D**). Taken together, these experiments suggest that the oxidative stress, mitochondrial dysfunction, and consequential cell death apparent in DS HNPs during development arise in part from soluble, secreted S100B and Aβ42 effects on the RAGE pathway.

## Discussion

While various HSA21 genes have been implicated in oxidative injury in DS, their cooperative interactions and consequent effects have not really been explored within neural progenitors during development and disease. Previous findings have suggested that APP and S100B may interact synergistically in contributing to the neuronal dysfunction and injury. Adult double transgenic (mutant APP (mAPP)/RAGE) mice demonstrated increased activation of stress pathways (phosphorylation of p38 and JNK) and altered expression of markers of synaptic plasticity (MAP kinases), leading to early abnormalities in spatial learning/memory [27]. Additionally, adult brains from double transgenic (Tg2576/APP-huS100B) mice displayed augmented reactive astrocytosis and microgliosis, high levels of S100 expression, and increased levels of proinflammatory cytokines [28]. Neuronal injury through APP is thought to enhance glial upregulation of S100B and secretion of soluble S100B, thereby promoting further neuronal injury. Increases in soluble APP, Aβ42 and Aβ42 antibody have been reported in the plasma of DS patients [29], while our prior studies have shown that soluble S100B induces p38 and JNK phosphorylation within DS neural progenitors [6]. The current studies now show a cooperative role for both these proteins in DS neural progenitor injury early in development. Both S100B and APP are constitutively overexpressed in DS HNPs, expression of each protein directly enhances expression of the other, and both proteins (S100B/A β42) appear to be secreted. The extracellular effects of APP appear to result, in part, from its processing to extracellular Aβ42 possibly through RAGE, which serves as a receptor for both S100B and Aβ42 [30]. Presumably, the trisomy of the S100B and APP, initiates a progressive cascade that further enhances the expression and secretion of these individual proteins, activates the RAGE-dependent pathway, and thereby additively promotes neural cell death.

It is still unclear as to whether the decreased neuronal numbers and hypotrophy in DS brains are due to increased cell death, decreased proliferation, or more likely, a combination of both. Several studies using early DS fetal brain samples and animal models find that decreased proliferation of neural progenitors in the ventricle or dentate gyrus play a dominant role in reducing neuronal production [2], [31], [32]. However, oxidative stress has been attributed to neuronal degeneration seen in later DS pathogenesis [33], [34]. Several studies using cultured DS neurons from fetal DS brain also support an early increase in oxidative stress leading to premature loss in neuronal viability [13], [35]. We have previously shown that constitutive overexpression of HSA21 associated S100B in human DS neural progenitors leads to increased levels of ROS and cell death in vitro [6], [9]. The current work extends these findings in demonstrating a synergistic effect between two HSA21 genes, S100B and APP, in promoting oxidative stress, and consequential cell death and gliosis both in vivo and in vitro. That said, we also have observed a reduction in HNPs proliferation in ventricular zone of DS frontal cortex (unpublished data). These observations are consistent with Guidi's report on the presence of both increased apoptosis and reduced proliferation in the dentate gyrus, hippocampus and parahippocampal gyrus of DS fetal brains [3]. Finally, multiple studies have suggested different mechanistic causes for the change in redox state in contributing to early neural pathological changes in fetal DS brain [36]. In one report, HSA21 associated SOD1 is increased in fetal DS brain [37]. In another report, the increased oxidative stress in fetal DS is suggested to be the consequence of low levels of reducing agents and enzymes involved in removal of hydrogen peroxide rather than overexpression of HSA21 associated SOD1 [38]. Overall, these collective studies would indicate some role for both oxidative stress and cell death, as well as a change in the proliferative rates of progenitor pool, in promoting the reduction in DS brain size.

Several studies suggest a primary role for Aβ42, as opposed to APP and Aβ40, in the pathogenesis of DS. Aβ42 deposition has been observed in the cerebral tissue of DS subjects [39]. Expression levels also appear earlier than Aβ40 accumulation [40]. Furthermore, increases in soluble APP, Aβ42 and Aβ42 antibody have been found in the plasma of DS patients [29]. Lastly, inhibition of Aβ42 production with a γ secretase inhibitor led to a reduction in APP induced neuronal apoptosis, which suggests that soluble APP and Aβ40 were not the primary cause of neurotoxicity [41]. That said, some primary role for either cytoplasmic or soluble APP or Aβ40 may still contribute to the DS phenotype and additional studies would be required to address these possibilities.

Human neural progenitors are more susceptible to S100B and Aβ42 mediated toxicity as compared to neurons or astrocytes. We observed cytotoxic effects of S100B and Aβ42 at lower nanomolar to picomolar concentrations. The soluble Aβ42 preparation used in this study includes not only the fibrillar form but also oligomers, which could be toxic to neurons in very low concentration [42], [43], [44]. However, S100B is generally thought to have a neuroprotective role at low concentrations within neurons or astrocytes, but at higher micromolar concentrations causes cell death [21], [45], [46]. The increased susceptibility of HNPs to lower concentrations of S100B and APP may partially be attributable to the fact that both proteins are already overexpressed within the same progenitor cells (**Figure 2A**) - as opposed to the neuronal (APP) and glial (S100B) paradigm seen in the adult brain. Moreover, S100B and APP appear to increase mitochondrial hydrogen peroxide levels directly, consistent with reports that APP/Aβ directly accumulate within and effect mitochondrial dynamics [47], [48]. Overall, the increased sensitivity of HNPs to these proteins would only further augment the pathological processes invoked from constitutive overexpression of S100B and APP in DS cells.

A gliocentric shift in the progenitor pool would provide a potential explanation for the preferential loss in later-born, presumptive GABA-ergic interneurons. Although cortical neuron density appears normal in early gestation DS brain, neuronal numbers decline at later (>23 weeks) gestational ages [49], [50]. Prior studies have suggested that HSA21-associated DYRK1A downregulates REST at a very early developmental stage, thereby causing a skewed ratio of primitive endoderm at the expense of neuroectodermal progenitors, leading to a reduction in neurogenesis [8]. This decline in neuronal production, however, would not necessarily explain the cell type and age specific loss in GABA-ergic neurons, as opposed to total neuronal numbers, in the DS cortex [14]. More recent studies have suggested impairments in interneuron development, potentially due to overexpression of a transcription factor gene, COUP-TF1/NR2F1, although the mechanism behind changes in this non-HSA21 associated transcription factor in effecting interneurons is not clear [7]. The current work now raises the possibility that ongoing neural progenitor cell death from oxidative stress enhances gliocentric progenitor characteristics at the expense of neuronal progenitor phenotypes. This shift becomes more prominent later in cortical development when the cyclical and synergistic roles played by such inflammatory mediators such as S100B and APP become more pronounced. Under this model, the loss in neuronogenic progenitors would manifest later in development and thereby effect interneuron production.

Several HSA21-dependent processes may contribute to the overall reduction in neuronal numbers in the cortices of individuals with DS. First, we find that HNPs are more susceptible to S100B and APP dependent mechanisms of cell injury and constitutive overexpression of these genes in DS HNPs leads to increased cell death. Second, inflammation as seen with induction of the stress response kinases is known to promote glial proliferation and reactive gliosis [51] and increased astrocytes or glial phenotypes have been reported in DS brain [3]. We have previously shown that S100B activates the JNK stress response pathways [6] and now observe an increase in glial characteristics within DS neural progenitors. This response to inflammatory mediators appears to enhance proliferation of gliocentric progenitors at the expense of neuronal progenitors. This finding occurs not only in HNPs *in vitro*, but also in TsDn65 or APP/S100B transgenic mice *in vivo*. Third, HSA21-associated genes may actually induce gliocentric phenotypes. For example, S100B is a glial marker that promotes glial phenotypes whereas HSA21-localized Olig2 is a transcription factor essential for development of oligodendrocytes. The number of Olig2(+) progenitors increases in the injured CNS, and Olig2(+) cells preferentially differentiate into GFAP-expressing astrocytes, the main contributors to glial scars which further secrete S100B [52], [53]. This sequence of events would further impair neuronogenesis. Fourth, glial progenitors may be more resistant to the increased oxidative stressor [6], and the increased ROS levels in DS may more readily compromise neuronogenic progenitors.

In this study, we have characterized the neuropathological phenotypes associated with early fetal DS cortex and HNPs. The findings of increased ROS, mitochondrial dysfunction, cell death and a glial shift in many ways mirrors the ongoing neurodegeneration and gliosis seen in the mature brain. Constitutively overexpressed, contiguous genes along HSA21, including S100B and APP, act in a synergistic manner to enhance secretion of these proteins and activate the RAGE cascade. This pathway induces mitochondrial hydrogen peroxide generation, loss of membrane potential, and ultimately causes cell death. The ongoing inflammatory and stress response furthermore encourages HNPs to adopt gliocentric characteristics, thereby not only enhancing this cycle of cell injury but also surprisingly, impairing neurogenesis. Finally, these studies suggest that the early defects in the progenitor pool caused by various HSA21 genes will likely alter the later stages of development - involving neuronal production and viability, thereby amplifying and augmenting the MR and AD seen in this disorder. More specifically, future studies will be directed toward addressing whether this shift in progenitor phenotype is responsible for the changes in proliferative rates observed between normal and DS brains.

## Materials and Methods

### Human tissue, ethical and licensing considerations

The study has been approved by the Institutional Review Board (IRB) at the Beth Israel Deaconess Medical Center (BIDMC) and Brigham and Women's Hospital. The de-identified human discarded tissue was obtained from pathological samples during autopsy. The tissue sections or neural progenitors from DS and age-matched control brains (14–21W GA) were used in this study for each of the experiments described. It is a discarded tissue protocol and is exempt from informed consent, as determined by the ethics and IRB review committee at BIDMC, and no informed consent was obtained. The approved protocol number is 2004-P-000299/5. The detail information of the tissues is listed in Table 1.

**Table 1:** Aborted fetal brain tissue used in this study.

Gestational Age	Karyotype	Gender	Postmortem Interval
14 weeks	46, XX	Female	3 hours
14 weeks	46, XX	Female	4 hours
14 weeks	46, XY	Male	2 hours
14 weeks	47, XX, +21	Female	2 hours
14 weeks	47, XX, +21	Female	3 hours
14 weeks	47, XY, +21	Male	3 hours
18 weeks	46, XY	Male	3 hours
18 weeks	46, XY	Male	3 hours
18 weeks	46, XY	Male	2 hours
18 weeks	46, XX	Female	3 hours
18 weeks	46, XX	Female	4 hours
18 weeks	46, XY, +21	Male	3 hours
18 weeks	46, XY, +21	Male	4 hours
18 weeks	46, XY, +21	Male	2 hours
18 weeks	46, XX, +21	Female	3 hours
18 weeks	46, XX, +21	Female	2 hours
21 weeks	46, XX	Female	4 hours
21 weeks	46, XX	Female	3 hours
21 weeks	46, XY	Male	3 hours
21 weeks	47, XX, +21	Female	3 hours
21 weeks	47, XX, +21	Female	2 hours
21 weeks	47, XY, +21	Male	3 hours

### Transgenic mice

Archival brain sections from 19 months old transgenic mice Tg2576 (APP over-expression) and Tg2576-huS100B (APP/S100B over-expression) were provided by Dr. Takashi Mori [28]. Trisomy16 mouse TsDn65 was obtained from The Jackson Laboratory (Bar Harbor, Maine, USA). P0 brains were fixed with 4%PFA overnight, and frozen after 20% sucrose infiltration overnight, 14 µm sections were collected for immunostaining. The animal studies have been approved by the Institutional Animal Care & Use Committee (IACUC) at the Beth Israel Deaconess Medical Center. The approved IACUC protocol number for animal work is 003-2011/100789.

### Antibodies and reagents

Antibodies used for immunostaining, ELISA and western blot analyses were as follows: Mouse anti-S100B (1∶100 for ELISA and 1∶500 for western blot, AbCam, Cambridge, UK), peroxidase-conjugated anti-S100B (1∶2000 for ELISA, AbCam, Cambridge, UK), rabbit anti-S100B (1∶200 for immunostaining and 1∶1000 for western blot, DAKO, Glostrup, Denmark), mouse anti-APP (1∶100 for immunostaining, Millipore, Billerica, MA, USA), rabbit anti-APP (1∶1000 for western blot, gift of Professor Sam Gandy), goat anti-Ephrin B2 (1∶100, R&D, Minneapolis, MN, USA), mouse anti-RAGE (1∶1000, R&D, Minneapolis, MN, USA), mouse anti-vinculin (1∶1000, AbCam, Cambridge, MA, USA), rabbit anti-GFAP (1∶500 for immunostaining, and 1∶2000 for western blot, DAKO, Glostrup, Denmark), mouse anti-MBP (1∶1000, Abcam, Cambridge, MA, USA), mouse anti-CNPase (1∶100, Millipore, Billerica, MA, USA), mouse anti-Pax6 (Millipore, Billerica, MA, USA), rabbit anti-olig2 (1∶1000, gift of Professor Charles Stiles, Dana-Farber Cancer Institute, Boston, MA, USA), mouse IgM anti-O1 and O4 (1∶50, gift of Professor Timothy Vartanian, Weill Cornell Medical College, New York, USA), mouse anti-MAP2 (1∶200 for immunostaining and 1∶1000 for western blot, Sigma, Saint Louis, MO, USA), rabbit anti-DCX (1∶200, gift of Professor Christopher Walsh, Children's Hospital, Boston, MA, USA), mouse anti-NeuN (1∶200, Millipore, Billerica, MA, USA), rabbit anti-GFP (1∶1000, Abcam, Cambridge, MA, USA), and rabbit anti-ZsGreen (1∶1000, Clontech, Mountain View, CA, USA). Reagents used for mitochondria and cell death studies are as follows: MitoTracker Deep Red (Invitrogen, Carlsbad, CA, USA), MitoPY1 (gift of Professor Christopher J. Chang, UC Berkeley, CA, USA), In situ Cell Death Detection Kit, TMR red (Roche Diognostics, Mannheim, Germany), Aβ42 (American Peptide, Sunnyvale, CA, USA), S100B (Calbiochem, San Diego, CA, USA), Aβ42 ELISA Kit (Invitrogen, Carlsbad, CA, USA), phenserine ((3aS,8aR)-1,2,3,3a,8,8a-Hexahydro-1,3a,8-trimethylpyrrolo[2,3-b]indol-5-ol5-(N-phenylcarbamate), Tocris, Ellisville, Missouri, USA) and RAGE antagonist dalteparin sodium (Dalteparin sodium, Pfizer Inc, New York, USA), IFN-γ was from Pharmingen (Becton Dickinson, Italy).

### Human neural progenitor cell cultures

Methods for VZ dissection and dissociation followed general guidelines described previously [6], [54]. In brief, samples were obtained along the periventricular zone within the frontal cortex, located by the landmark of Sylvian fissure and central fissure, minced and washed in cold Hank's buffered saline solution and mechanically dissociated with pipettes. The samples were then strained through a 40-µm cell strainer (Falcon, San Jose, CA, USA). The dissociated cells were spun down, the media aspirated and cells were placed in at low dilution (1×10^6^ per 5 ml) in neurosphere medium (StemPro NSC SFM, Invitrogen, Carlsbad, CA, USA) for expansion. The cultures were maintained in a 37°C/5% CO2 incubator for 1 to 2 weeks before the analysis. To initiate differentiation, dissociated cells were plated on poly-D-lysine/laminin 1-coated coverslips at a density of 1×10^5^ cells per coverslip (24 mm×24 mm). Oligodendrocyte differentiation was achieved by maintaining the cells in KNOCKOUT™DMEM/F12 (Invitrogen, Main, MD)+2% B27(50×, Invitrogen, Main, MD)+10 ng/ml bFGF (R&D, Minneapolis, MN, USA)+100 ng/ml SHH (R&D, Minneapolis, MN, USA)+10 ng/ml PDGF-AA (R&D, Minneapolis, MN, USA) for 2 days, then switching to the same medium without growth factors for another 5 days. Neuronal differentiation was achieved by maintaining cells in KNOCKOUT™DMEM/F12 +2% B27 (50×) for 7 days. Astrocyte differentiation was done by culturing cells in KNOCKOUT™DMEM/F12 +1%FBS for a week. The pharmacological treatments were in the same neurosphere medium without EGF and bFGF. The preparation of Aβ42 was as follows, Aβ42 was dissolved with distilled water to make 10 µg/ml stock solutions, and put into 37°C incubator for 24 h before further dilution and use.

### Constructs, viral production and transfection or infection

pEGFP-C1-S100B and pHAGE-CMV-MCS-IZsGreenW-APP constructs were made for *in vitro* overexpression experiments. Human S100B full-length cDNA sequence was cloned into pEGFP-C1 vector with XhoI/Hind III cutting site. The transfection of pEGFP-C1-S100B construct in dissociated neural progenitors was performed using Transfectin (Bio-Rad, Hercules, CA, USA) according to the company's product instruction. Human APP full length cDNA sequence were cloned into pHAGE-CMV-MCS-IZsGreenW vector (gift of *The Harvard Gene Therapy Initiative*) with NotI/XbaI cutting site; Production of lentiviruses was done in 293T cells as described by Richard Mulligan's lab [55], [56], the MOI of 1 is used for both viruses. Dissociated neural progenitor cells were infected with lentivirus or transfected with constructs carrying the target genes, and kept in neural stem cell medium for 4 days before analysis.

### Immuno-staining and TUNEL analyses

Tissue sections after antigen retrieval or fixed cells were placed in blocking solution with PBS containing 3% goat serum, incubated overnight in the appropriate antibody, and processed through standard fluorescent secondaries (CY2, CY3, Jackson Immunoresearch Laboratories, Westgrove, PA, USA, and FITC, Sigma, 1∶500). Specimens were examined using confocal fluorescence microscopy after mounting in appropriate media. Apoptosis were detected in sections or neurospheres by TUNEL using In Situ Cell Death Detection Kit, TMR red (Roche). Sections with positive stained cells were counted in at least three sections for each patient and 4 patients for each assay. Cells staining positive for expressed markers were counted in five randomly chosen microscopic fields (0.02 mm^2^; magnification: 630×) along the ventricular lining in each object; 5–10 neurospheres were sampled on each treatment or patient, the TUNEL positive cells were counted against the total cells in the field randomly selected. TUNEL positive cells and immuno-positive cells in mouse sections were counted in cortex or dentate gyrus area (0.02 mm^2^; magnification: 630×) of equal location, at least three sections for each mouse and three mice for each group.

### Western blot

Proteins were extracted from neurospheres or cell lines by previously described methods [6], [9]. Briefly, cells were solubilized in lysis buffer, separated on a 7.5% SDS–PAGE gel and transferred onto PVDF membrane. The membrane was probed with the appropriate antibody and detected by enhanced chemiluminescence.

### ELISA assay

Enzyme-linked immunosorbent assay (ELISA) for S100B was carried out on tissue supernatants as well as in the cell lysates. Briefly, 15 µL of sample plus 15 µL of Tris buffer were applied on a microtitre plate previously coated with monoclonal anti-S100B (1∶1000; AbCam) in carbonate buffer and blocked with 1% bovine serum albumin. After washing, peroxidase-conjugated anti-S100B (1∶2000; AbCam) was added and incubation continued for 1 h. The plate was washed, 0.2 mL of peroxidase substrate (Fast OPD; Sigma, Milan, Italy) was added and the plate was incubated for a further 30-min period in the dark. Absorbance was measured at 450 nm on a microtitre plate reader. S100B levels in the samples were determined using a standard curve of S100B and expressed as ng/mL. Aβ42 ELISA was carried out with Aβ42 ELISA kit (Invitrogen, Carlsbad, CA, USA) following the company's protocol.

### Nitric oxide assay

Nitric Oxide (NO) production was measured as the stable metabolite nitrite (NO_2_^−^) accumulated in the incubation medium of neural progenitor cells after 24 hours following LPS + IFN-γ addition, using a spectrophotometric assay based on Griess reaction as previously [57].

### MitoTracker deep red and MitoPY1 assay

The neurospheres or attached neural progenitor cells after treatments were incubated with 0.5 µM MitoTracker deep red (Invitrogen M22426, Carlsbad, CA, USA) together with 5 µM MitoPY1 for 30 min in 37°C/5% CO_2_ incubator. MitoPY1 is a mitochondrial-targeted fluorescent probe that responds to hydrogen peroxide by a turn-on increase in fluorescence intensity [15]. The cells were then fixed with 4% paraformaldehyde for 10 min, and wash with PBS, counterstained with Hoechst 33342 (Invitrogen, Carlsbad, CA, USA). Cells were mounted on slides and scanned by fluorescence microscope. The intensity of staining was measured in 10 randomly chosen cells from each of nine neurospheres or wells by each treatment. The numbers of MitoPY1 stained granules were counted in each case.

### Statistical analyses

Results were expressed as the mean +/− STDEV of n experiments. Statistical analysis was performed with Student's T test or one way ANOVA, with P<0.05 considered significant.

## Supporting Information

Figure S1 **Gliocentric cell fate shift within human fetal DS HNPs.** Immunostaining of WT and DS HNPs show shifted cell fates after differentiation for 1 week. The glial cells are stained with O1 (rhodamine), O4 (rhodamine), CNPase (fluorescein), S100B (fluorescein), MBP (rhodamine), Olig2 (fluorescein) and GFAP (rhodamine); the neuronal cells are stained with MAP2 (fluorescein), DCX (fluorescein) and NeuN (fluorescein). The quantification graph showing decreased neuronal cells and increased glial cells in DS HNPs differentiation compared to WT controls are showed to the right. Scale bar is 25 µm. Data are represented as mean +/− STDEV, ** p-value<0.01, *** p-value<0.001 by two tailed t-test.
(TIF)

Figure S2 **Intracellular over-expression of S100B and APP cause loss of mitochondrial membrane potential.** (A) APP-lentiviral infection of WT HNPs dose-dependently decreases MitoTracker deep red (rhodamine) intensities 2 days after infection, with infected cells in fluorescein. The increased expression of APP and secretion of Aβ42 are showed on the right by western blot and ELISA. (B) EGFP-S100B transfection of WT HNPs shows a similar pattern as that in APP-lentiviral infections 48 hours after transfection. The increased expression and secretion of S100B are showed to the right by western blot and ELISA. (C) A combination of APP-lentiviral infection and EGFP-S100B transfection for 2 days in HNPs shows an additive effect in reducing the mitochondrial membrane potential. Scale bars are 25 µm in A, B and C; data are represented as mean +/− STDEV, * p-value<0.05, *** p-value<0.001 by two tailed t-test and one-way ANOVA.
(TIF)

Figure S3 **Intracellular over-expression of S100B and APP cause increased apoptosis in HNPs and transgenic mice.** (A) APP-lentiviral infection of WT HNPs dose-dependently increases TUNEL positive staining (rhodamine) 4 days after infection, with infected cells in fluorescein. Graphical quantification is to the right. (B) EGFP-S100B transfection of WT HNPs shows a similar pattern as that in APP-lentiviral infection 4 days after transfection. Graphical quantification is to the right. (C) A combination of APP-lentiviral infection and EGFP-S100B transfection in WT HNPs for 4 days shows an additive effect of increasing apoptosis. (D) TUNEL staining with detection under rhodamine fluorescence shows increased labeling of cells in the frontal cortex of Ts65Dn mice compared to WT control (left panel, n = 3 for each group of mouse). The increased TUNEL labeling of cells are also found in the subgranular zone of dentate gyrus of 19 months old APP (Tg2576) or APP/S100B (Tg2576-huS100B) over-expression mice compared to WT control (right panel, n = 4 for each group of mouse). The quantification graphs are below. (E) Quantification graphs show additive effects of S100B and Aβ42 in enhancing the observed mitochondrial dysfunction 24 hours after treatment (**Figure 3B**). Scale bars are 200 µm for low magnification and 25 µm for high magnification in A, B, C and D; data are represented as mean +/− STDEV, * p-value<0.05, ** p-value<0.01, *** p-value<0.001 by two tailed t-test and one-way ANOVA.
(TIF)

Figure S4 **Intracellular over-expression of S100B and APP promote gliocentric phenotypes.** (A) Fluorescent photomicrographs in the cortex of early postnatal (P0) Ts65Dn mice show increased numbers of immunostaining on glial markers such as S100B (fluorescein, upper left panel), GFAP (rhodamine, upper right panel) and PDGFRA (rhodamine, lower left panel) compared to WT controls. There is also a decreased numbers of immunostaining on neuronal marker MAP2 (rhodamine, lower right panel) in Ts65Dn mice compared to WT controls. The white arrowheads in low magnification figures mark the VZ in frontal cortex; the high magnification figures show cells in VZ except for MAP2 in cortical plate. (B) Fluorescent photomicrographs of S100B (rhodamine, left panel) and GFAP (rhodamine, right panel) staining (counterstained with Hoechst 33342) in the cortex of 19 months old mice show increased apoptosis and gliosis in APP (Tg2576) or APP/S100B (Tg2576-huS100B) over-expressing mice compared to WT control. Increased rhodamine stained cells are counted in the subgranular zone of dentate gyrus, with the quantification of immuno-positive cells showed below (n = 4 for each group of mice). (C) Fluorescent photomicrographs of CNPase (rhodamine, left panel) and myelin basic protein (MBP, rhodamine, right panel) staining (counterstained with Hoechst33342) in the cortex of 19 months old mice shows increased expression of two oligodendrocyte markers in the APP (Tg2576) or APP/S100B (Tg2576-huS100B) over-expressing mice compared to WT control. Intense rhodamine fluorescence is seen in the subcortex and subhippocampus (n = 4 for each group of mice). Scale bars are 200 µm for low magnification and 25 µm for high magnification in A, B and C.
(TIF)

Figure S5 **RAGE blocking and APP inhibition synergistically reduce oxidative stress and apoptosis.** (A) Photographs show the S100B dose dependently increase H_2_O_2_ production shown by MitoPY1 staining (fluorescein) and decrease mitochondrial membrane potential shown by MitoTracker deep red staining (rhodamine), which can be blocked by RAGE antibody or dalteparin sodium after 24 hours. (B) The large numbers of TUNEL+ cells in DS HNPs decrease to normal level after Anti-RAGE antibody or dalteparin sodium treatment for 24 hours. (C) Fluorescent photomicrographs of DS HNPs show a dose-dependent rise in mitochondrial membrane potential, as evidenced by an increase in MitoTracker deep red staining (rhodamine) after 24 hours treatment with RAGE and APP inhibitors. A corresponding decrease in mitochondrial hydrogen peroxide levels is also apparent, as evidenced by MitoPY1 staining (fluorescein). (D) The number of TUNEL positive, DS HNPs are decreased after pretreatment with the RAGE antagonist dalteparin sodium, APP inhibitor phenserine, or both (dalteparin sodium + phenserine) for 24 hours. Scale bars are 25 µm in A and C, 200 µm for low magnification and 25 µm for high magnification in B and D.
(TIF)
