# Supplementary figures and images for: S100B and APP Promote a Gliocentric Shift and Impaired Neurogenesis in Down Syndrome Neural Progenitors
Source: PLoS One. 2011 Jul 11;6(7):e22126. doi: 10.1371/journal.pone.0022126 (PMC3133657; doi:10.1371/journal.pone.0022126)

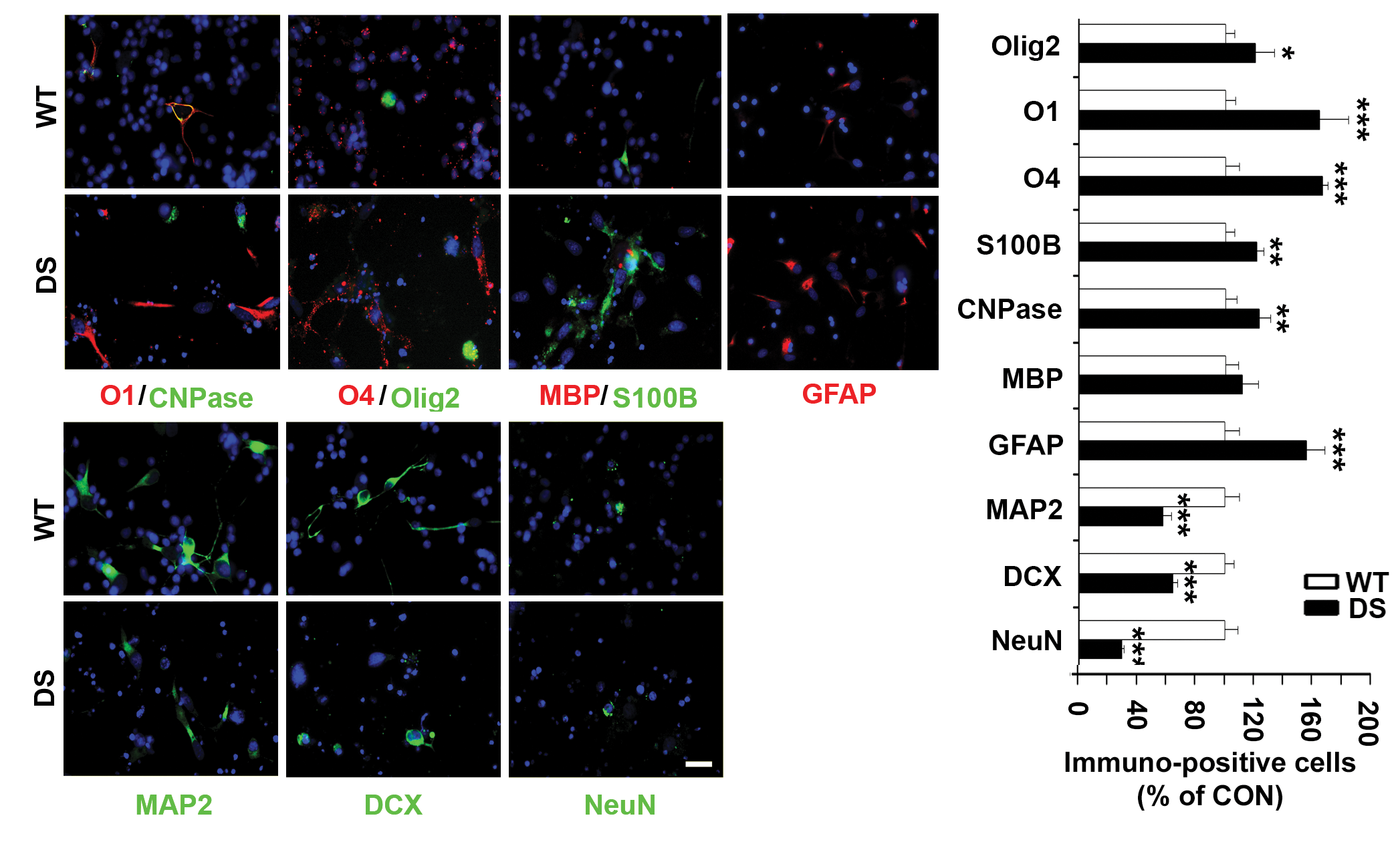

Supplement: Figure S1 — Gliocentric cell fate shift within human fetal DS HNPs. Immunostaining of WT and DS HNPs show shifted cell fates after differentiation for 1 week. The glial cells are stained with O1 (rhodamine), O4 (rhodamine), CNPase (fluorescein), S100B (fluorescein), MBP (rhodamine), Olig2 (fluorescein) and GFAP (rhodamine); the neuronal cells are stained with MAP2 (fluorescein), DCX (fluorescein) and NeuN (fluorescein). The quantification graph showing decreased neuronal cells and increased glial cells in DS HNPs differentiation compared to WT controls are showed to the right. Scale bar is 25 µm. Data are represented as mean +/− STDEV, ** p-value<0.01, *** p-value<0.001 by two tailed t-test. (TIF) [file pone.0022126.s001.tif]

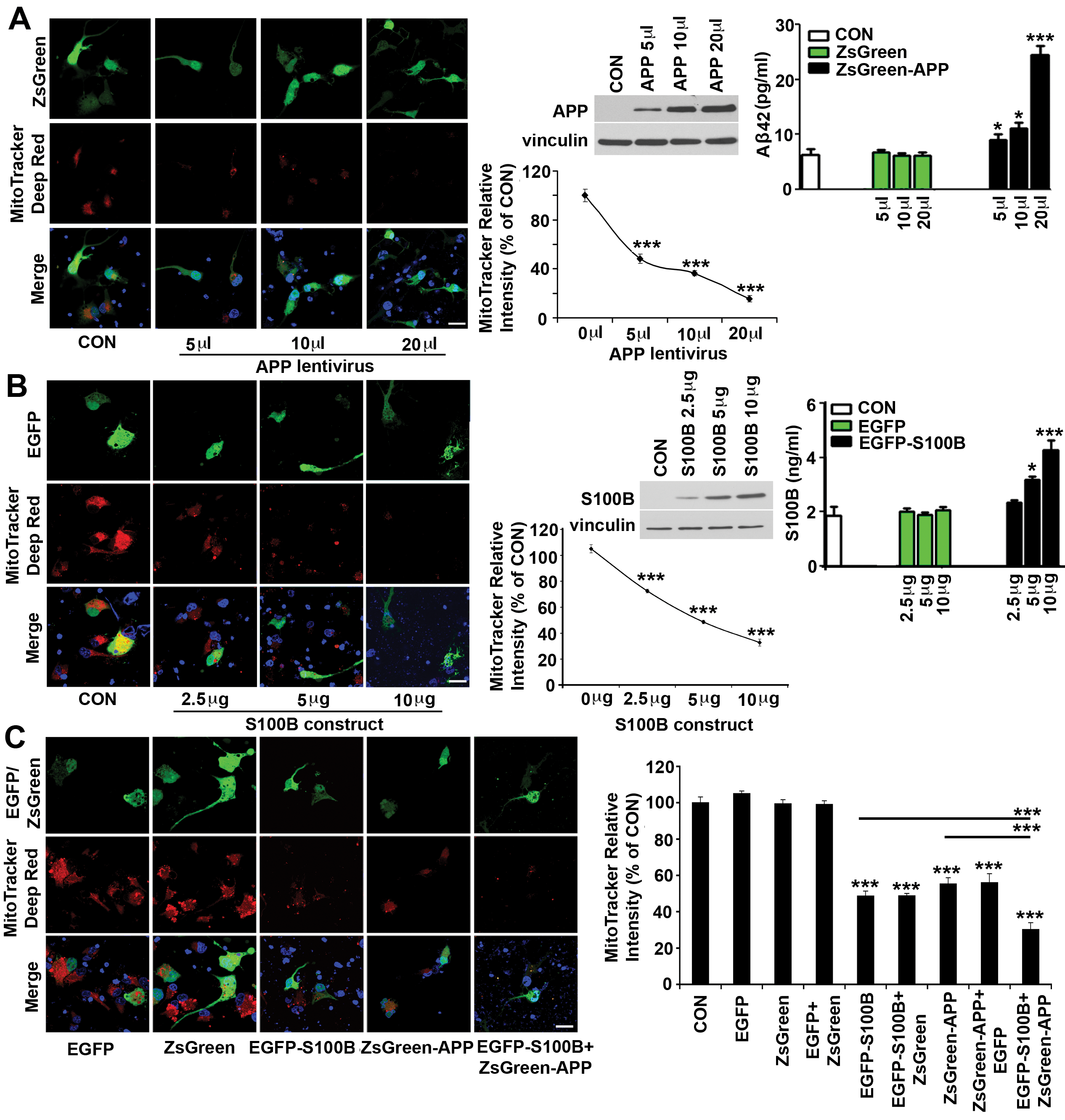

Supplement: Figure S2 — Intracellular over-expression of S100B and APP cause loss of mitochondrial membrane potential. (A) APP-lentiviral infection of WT HNPs dose-dependently decreases MitoTracker deep red (rhodamine) intensities 2 days after infection, with infected cells in fluorescein. The increased expression of APP and secretion of Aβ42 are showed on the right by western blot and ELISA. (B) EGFP-S100B transfection of WT HNPs shows a similar pattern as that in APP-lentiviral infections 48 hours after transfection. The increased expression and secretion of S100B are showed to the right by western blot and ELISA. (C) A combination of APP-lentiviral infection and EGFP-S100B transfection for 2 days in HNPs shows an additive effect in reducing the mitochondrial membrane potential. Scale bars are 25 µm in A, B and C; data are represented as mean +/− STDEV, * p-value<0.05, *** p-value<0.001 by two tailed t-test and one-way ANOVA. (TIF) [file pone.0022126.s002.tif]

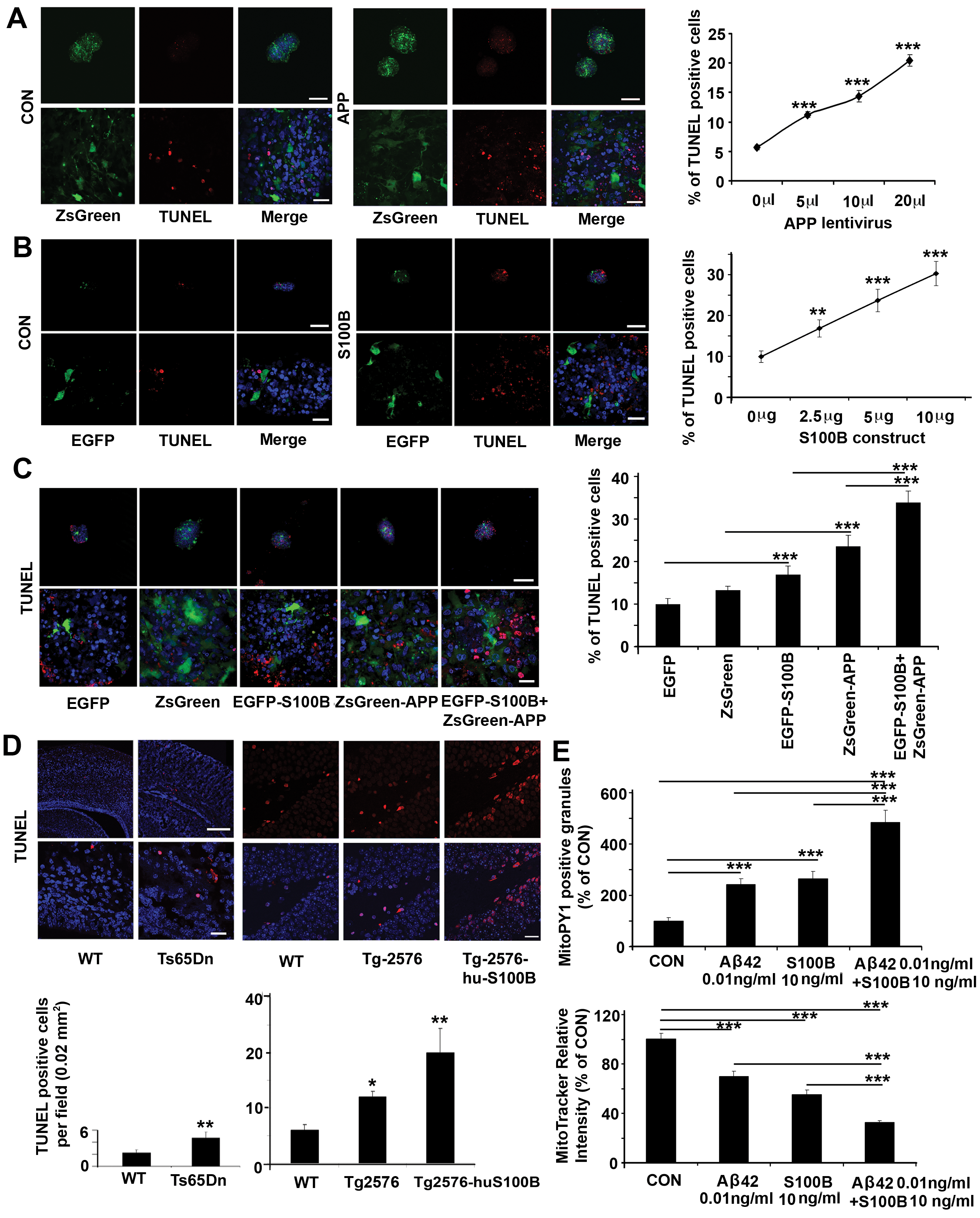

Supplement: Figure S3 — Intracellular over-expression of S100B and APP cause increased apoptosis in HNPs and transgenic mice. (A) APP-lentiviral infection of WT HNPs dose-dependently increases TUNEL positive staining (rhodamine) 4 days after infection, with infected cells in fluorescein. Graphical quantification is to the right. (B) EGFP-S100B transfection of WT HNPs shows a similar pattern as that in APP-lentiviral infection 4 days after transfection. Graphical quantification is to the right. (C) A combination of APP-lentiviral infection and EGFP-S100B transfection in WT HNPs for 4 days shows an additive effect of increasing apoptosis. (D) TUNEL staining with detection under rhodamine fluorescence shows increased labeling of cells in the frontal cortex of Ts65Dn mice compared to WT control (left panel, n = 3 for each group of mouse). The increased TUNEL labeling of cells are also found in the subgranular zone of dentate gyrus of 19 months old APP (Tg2576) or APP/S100B (Tg2576-huS100B) over-expression mice compared to WT control (right panel, n = 4 for each group of mouse). The quantification graphs are below. (E) Quantification graphs show additive effects of S100B and Aβ42 in enhancing the observed mitochondrial dysfunction 24 hours after treatment ( Figure 3B ). Scale bars are 200 µm for low magnification and 25 µm for high magnification in A, B, C and D; data are represented as mean +/− STDEV, * p-value<0.05, ** p-value<0.01, *** p-value<0.001 by two tailed t-test and one-way ANOVA. (TIF) [file pone.0022126.s003.tif]

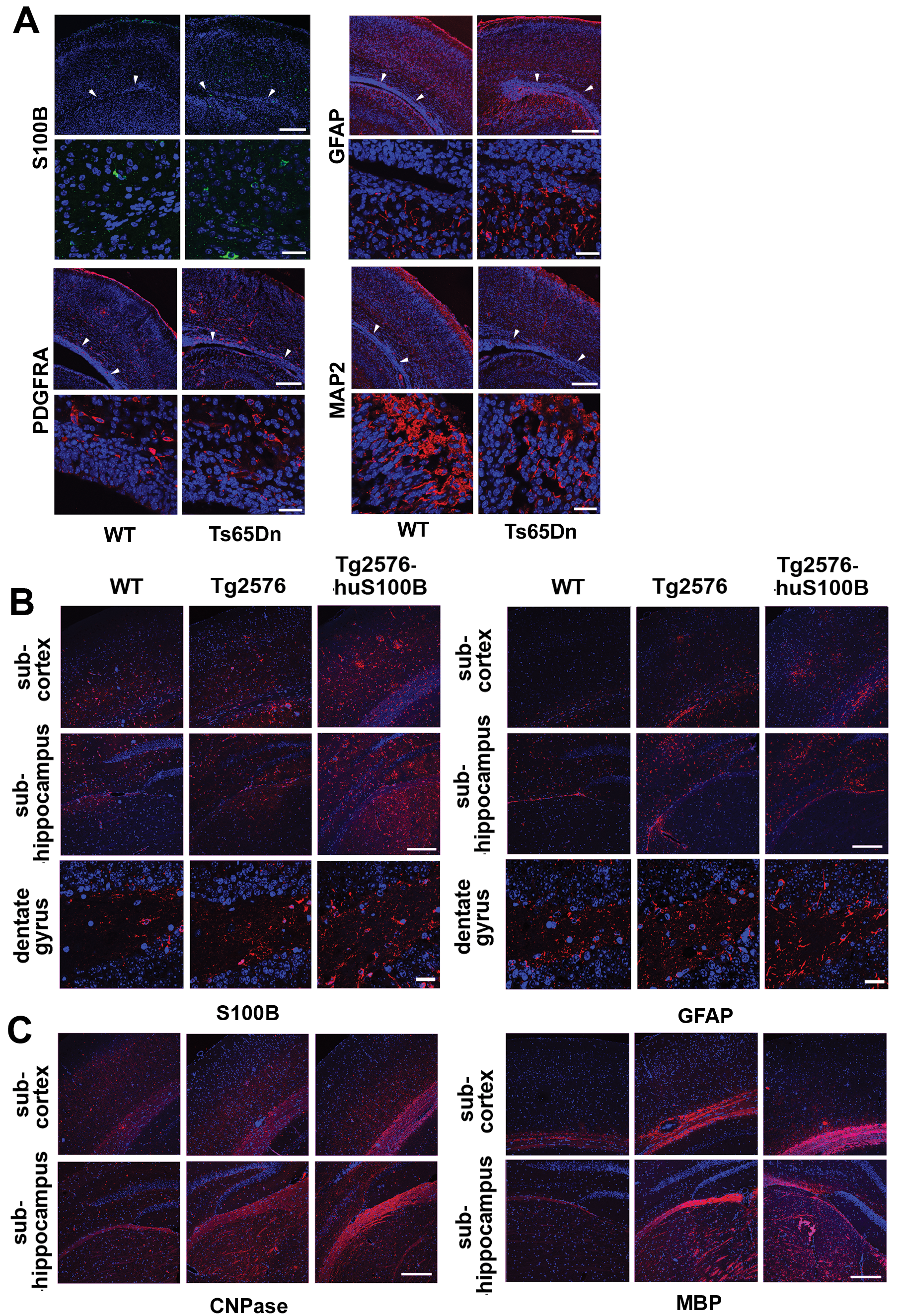

Supplement: Figure S4 — Intracellular over-expression of S100B and APP promote gliocentric phenotypes. (A) Fluorescent photomicrographs in the cortex of early postnatal (P0) Ts65Dn mice show increased numbers of immunostaining on glial markers such as S100B (fluorescein, upper left panel), GFAP (rhodamine, upper right panel) and PDGFRA (rhodamine, lower left panel) compared to WT controls. There is also a decreased numbers of immunostaining on neuronal marker MAP2 (rhodamine, lower right panel) in Ts65Dn mice compared to WT controls. The white arrowheads in low magnification figures mark the VZ in frontal cortex; the high magnification figures show cells in VZ except for MAP2 in cortical plate. (B) Fluorescent photomicrographs of S100B (rhodamine, left panel) and GFAP (rhodamine, right panel) staining (counterstained with Hoechst 33342) in the cortex of 19 months old mice show increased apoptosis and gliosis in APP (Tg2576) or APP/S100B (Tg2576-huS100B) over-expressing mice compared to WT control. Increased rhodamine stained cells are counted in the subgranular zone of dentate gyrus, with the quantification of immuno-positive cells showed below (n = 4 for each group of mice). (C) Fluorescent photomicrographs of CNPase (rhodamine, left panel) and myelin basic protein (MBP, rhodamine, right panel) staining (counterstained with Hoechst33342) in the cortex of 19 months old mice shows increased expression of two oligodendrocyte markers in the APP (Tg2576) or APP/S100B (Tg2576-huS100B) over-expressing mice compared to WT control. Intense rhodamine fluorescence is seen in the subcortex and subhippocampus (n = 4 for each group of mice). Scale bars are 200 µm for low magnification and 25 µm for high magnification in A, B and C. (TIF) [file pone.0022126.s004.tif]

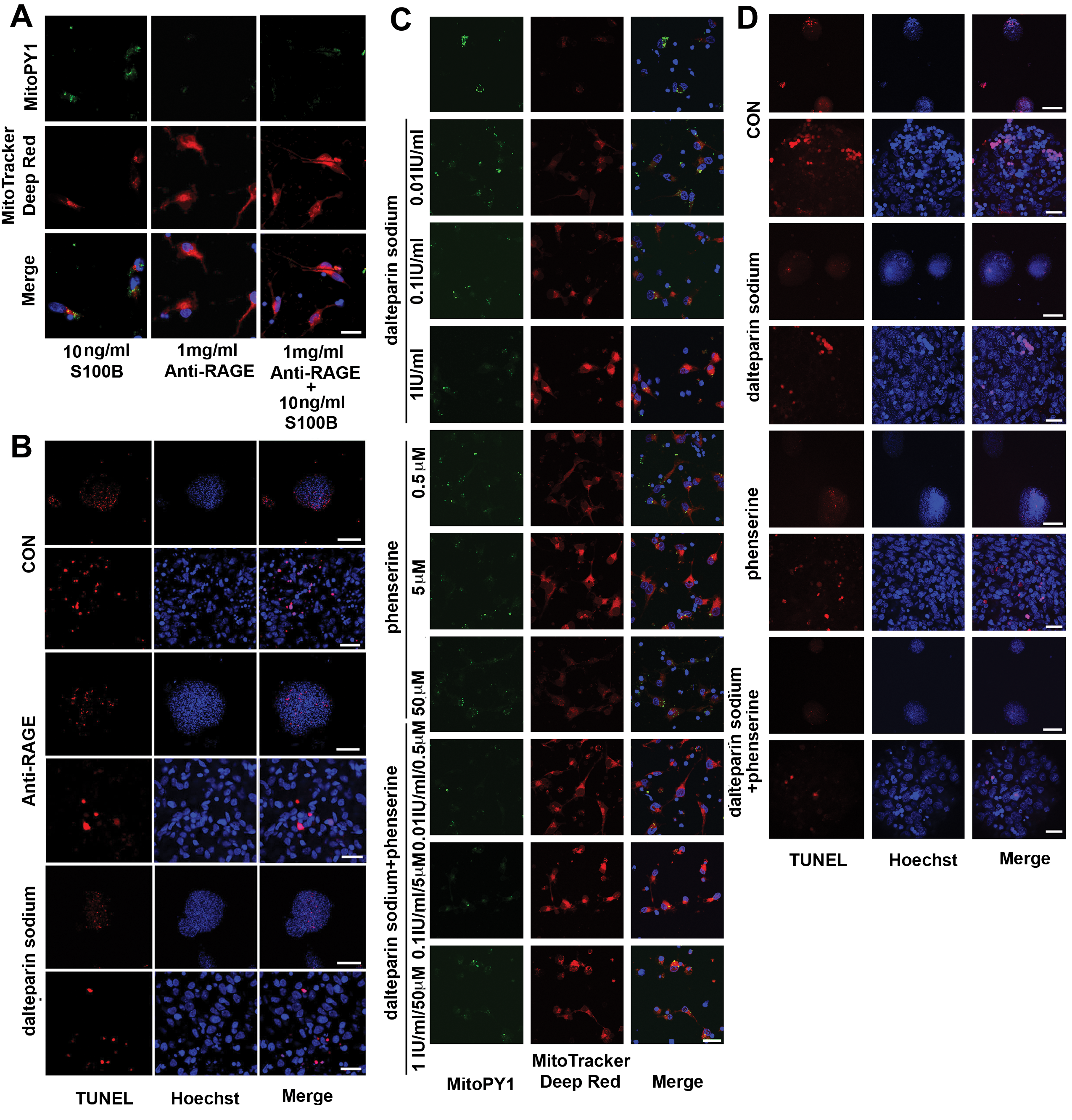

Supplement: Figure S5 — RAGE blocking and APP inhibition synergistically reduce oxidative stress and apoptosis. (A) Photographs show the S100B dose dependently increase H2O2 production shown by MitoPY1 staining (fluorescein) and decrease mitochondrial membrane potential shown by MitoTracker deep red staining (rhodamine), which can be blocked by RAGE antibody or dalteparin sodium after 24 hours. (B) The large numbers of TUNEL+ cells in DS HNPs decrease to normal level after Anti-RAGE antibody or dalteparin sodium treatment for 24 hours. (C) Fluorescent photomicrographs of DS HNPs show a dose-dependent rise in mitochondrial membrane potential, as evidenced by an increase in MitoTracker deep red staining (rhodamine) after 24 hours treatment with RAGE and APP inhibitors. A corresponding decrease in mitochondrial hydrogen peroxide levels is also apparent, as evidenced by MitoPY1 staining (fluorescein). (D) The number of TUNEL positive, DS HNPs are decreased after pretreatment with the RAGE antagonist dalteparin sodium, APP inhibitor phenserine, or both (dalteparin sodium + phenserine) for 24 hours. Scale bars are 25 µm in A and C, 200 µm for low magnification and 25 µm for high magnification in B and D. (TIF) [file pone.0022126.s005.tif]
